# Development and Internal Validation of a Prediction Model Incorporating Respiratory Muscle Function for CAT Score Deterioration in Stable COPD

**DOI:** 10.1111/crj.70234

**Published:** 2026-10-15

**Authors:** Muye Niu, Changye Wu, Shaoting Geng, Mian Song, Mingchao Cao

**Affiliations:** ^1^ Hebei Sport University Shijiazhuang Hebei China; ^2^ Department of Medicine Shijiazhuang Engineering Vocational College Shijiazhuang Hebei China; ^3^ Department of Otolaryngology Head and Neck Surgery, the First Ward The Second Hospital of Hebei Medical University Shijiazhuang Hebei China; ^4^ Department of Rehabilitation Medicine I The Second Hospital of Hebei Medical University Shijiazhuang Hebei China

**Keywords:** chronic obstructive pulmonary disease, COPD assessment test, nomogram, prediction model, respiratory muscle function

## Abstract

**Objective:**

The objectives are to investigate the association between respiratory muscle function and CAT score deterioration in stable chronic obstructive pulmonary disease (COPD) and to develop an internally validated prediction model.

**Methods:**

This single‐center retrospective cohort study included 203 patients with stable COPD. Clinically significant CAT score deterioration was defined as an increase of ≥ 2 points from baseline within 12 months. Predictors were selected using LASSO Cox regression and incorporated into a multivariable Cox model. Model performance and the incremental value of maximal inspiratory pressure (MIP) and diaphragm thickening fraction (DTF) were evaluated.

**Results:**

During follow‐up, 62 patients (30.5%) experienced CAT score deterioration. Higher FEV_1_% predicted (HR = 0.973, 95% CI: 0.956–0.990), MIP (HR = 0.974, 95% CI: 0.957–0.992), and DTF (HR = 0.908, 95% CI: 0.853–0.967) were associated with a lower risk, whereas higher baseline CAT scores and more frequent prior exacerbations were associated with a higher risk. The model showed an apparent 12‐month AUC of 0.873 (95% CI: 0.822–0.924), with an optimism‐corrected AUC of 0.859. Adding MIP and DTF increased the 12‐month AUC from 0.801 to 0.873 (ΔAUC = 0.072), with an NRI of 0.527 and an IDI of 0.058. Sensitivity analyses yielded consistent results.

**Conclusions:**

Lower MIP and DTF were independently associated with a higher risk of CAT score deterioration in stable COPD. Incorporating these parameters provided incremental predictive information beyond conventional clinical predictors. External validation is required.

## Graphical abstract

Lower maximal inspiratory pressure (MIP) and diaphragm thickening fraction (DTF) were independently associated with a greater risk of clinically significant CAT score deterioration over 12 months in patients with stable COPD. Adding MIP and DTF to conventional clinical predictors improved model discrimination, with the AUC increasing from 0.801 to 0.873. These findings highlight the potential value of respiratory muscle function for risk stratification in stable COPD.

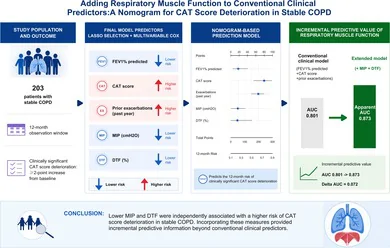

## 1 Introduction

Chronic obstructive pulmonary disease (COPD) is a chronic respiratory condition whose disease burden is largely manifested through reduced activity tolerance, increased symptom burden, and progressive deterioration in health‐related quality of life (HRQoL) [1, 2]. In recent years, HRQoL has emerged as a critical patient‐reported outcome in COPD management. Previous studies have indicated that deterioration in quality of life (QoL) is associated with adverse clinical outcomes, including exacerbations, hospitalizations, and mortality [3, 4]. Thus, identifying patients with stable COPD who are at risk of subsequent deterioration in health status is important for optimizing long‐term management and enabling early intervention.

Currently, assessment of future health‐status deterioration in patients with stable COPD relies predominantly on conventional clinical characteristics, including pulmonary function indices, symptom scores, and prior exacerbation history [5]. Among these, the forced expiratory volume (FEV) in 1 s as a percentage of predicted value (FEV_1_% predicted), COPD assessment test (CAT) score, and exacerbation frequency are widely employed for disease severity evaluation and prognostic determination [6]. However, in clinical practice, patients with comparable degrees of pulmonary function impairment often exhibit markedly divergent trajectories in QoL [7]. This observation suggests that airflow limitation measures alone may not adequately capture the factors associated with subsequent health‐status deterioration, highlighting the need to explore predictive indicators that complement traditional assessment frameworks.

Respiratory muscle dysfunction represents a common extrapulmonary manifestation of COPD. Chronic airflow limitation, pulmonary hyperinflation, and systemic inflammation are closely associated with increased respiratory muscle load and diminished functional capacity, which may be accompanied by reduced ventilatory efficiency, limited exercise tolerance, and exacerbated dyspnea [8]. Unlike conventional pulmonary function indices that primarily reflect airflow obstruction, respiratory muscle function parameters may, to some extent, reflect the functional reserve that patients retain for sustaining daily activities and coping with physiological challenges, potentially offering information distinct from that provided by traditional spirometric measures for QoL risk assessment [9]. Maximal inspiratory/expiratory pressure (MIP/MEP) is a non‐invasive index for evaluating respiratory muscle strength [10]. The recent development of bedside ultrasound technology has rendered diaphragmatic function assessment more accessible, with the diaphragm thickening fraction (DTF) considered a reliable indicator of active diaphragmatic contractile capacity [11]. Although previous studies have demonstrated associations between these parameters and reduced exercise tolerance, greater dyspnea, and increased exacerbation risk, evidence regarding their longitudinal predictive value for subsequent CAT score deterioration in patients with stable COPD remains limited. Furthermore, whether respiratory muscle function parameters provide incremental predictive information beyond conventional clinical factors remains unclear.

This retrospective cohort study was designed to use clinically significant CAT score deterioration within 12 months as the endpoint in patients with stable COPD. Candidate predictors were screened using LASSO Cox regression, and a nomogram integrating conventional clinical characteristics with respiratory muscle function parameters was constructed and internally validated. The incremental predictive value of respiratory muscle function parameters was further assessed via net reclassification improvement (NRI) and integrated discrimination improvement (IDI), with the aim of providing additional information for risk stratification in stable COPD.

## 2 Materials and Methods

### 2.1 Subjects

In this single‐center, retrospective cohort study, we retrospectively identified patients with stable COPD who had undergone respiratory muscle function testing and diaphragmatic ultrasonography at our hospital between January 2023 and January 2025. The protocol adhered to the *Declaration of Helsinki* and was approved by the Ethics Committee of the Second Hospital of Hebei Medical University (Approval No.: 2025‐R391‐N1). Due to the retrospective nature of the study and the use of anonymized clinical data, the requirement for informed consent was waived.

Inclusion criteria included: (1) age ≥ 40 years; (2) diagnosis of COPD according to the GOLD guidelines, with clinical stability at baseline, defined as no COPD exacerbation during the preceding 4 weeks and no change in the long‐term maintenance medication regimen during the same period; (3) completion of a comprehensive respiratory muscle function assessment at baseline, including MIP, MEP, and diaphragmatic ultrasonography; (4) independent completion of the CAT questionnaire; and (5) complete baseline clinical characteristic data.

Exclusion criteria were (1) coexisting major pulmonary diseases, including asthma, bronchiectasis, or severe interstitial lung disease; (2) overt psychiatric disorders or cognitive impairment that precluded reliable completion of the CAT; and (3) missing key baseline clinical or respiratory muscle function data.

A total of 267 potentially eligible patients were initially screened. Of these, 64 were excluded for the following primary reasons: 12 did not meet the definition of clinical stability at baseline, 11 had major coexisting pulmonary diseases, 4 had cognitive or psychiatric conditions that precluded reliable CAT assessment, 15 had incomplete baseline clinical or CAT data, and 22 had incomplete respiratory muscle function or diaphragmatic ultrasound data. The final study cohort comprised 203 patients.

### 2.2 Data Collection

Baseline data for all candidate predictor variables were obtained from the electronic medical record (EMR) system and encompassed four domains: (1) Demographics: age, sex, body mass index (BMI), and smoking history. (2) COPD‐related clinical features: COPD duration (years), baseline pulmonary function indices (FEV_1_% predicted and FEV_1_/forced vital capacity [FVC] ratio). According to medical history, the number of moderate‐to‐severe exacerbations in the 12 months before baseline was categorized as 0, 1, or ≥ 2 episodes. Moderate exacerbations were those with acute symptom worsening requiring systemic corticosteroids/antibiotics, and severe exacerbations were those resulting in emergency department consultation or hospitalization. Based on explicit records of pulmonary rehabilitation participation in the EMRs during the 12 months before baseline, patients were classified as having either a history of regular pulmonary rehabilitation or no such history. Baseline CAT scores were also recorded. (3) MIP and MEP were measured using a MasterScreen PFT system (Jaeger, Germany) following standardized respiratory muscle testing procedures. The equipment was routinely calibrated according to the manufacturer's recommendations. Patients were examined in the seated position using a mouthpiece and nose clip. MIP was measured from residual volume and MEP from total lung capacity. At least three acceptable maximal efforts were performed, and measurements were considered reproducible when the two highest values differed by no more than 10%. The highest value was used for analysis. MIP and MEP were expressed as absolute pressures (cmH_2_O); therefore, no reference‐equation‐based percentage‐predicted transformation was applied. Diaphragmatic morphology and function were assessed using a Clover 60 ultrasound system (Shenzhen Huasheng Medical Technology Co. Ltd., Shenzhen, China) equipped with a 7‐ to 10‐MHz linear‐array transducer. Patients were examined in the supine position during quiet tidal breathing. Diaphragm thickness was measured at the zone of apposition of the right hemidiaphragm, typically through the 8th–10th intercostal space between the anterior and midaxillary lines, using two‐dimensional B‐mode imaging. End‐expiratory and end‐inspiratory diaphragm thicknesses were measured over three consecutive respiratory cycles, and the mean values were used for analysis. DTF (%) was calculated as ([end‐inspiratory thickness − end‐expiratory thickness]/end‐expiratory thickness) × 100. Ultrasound examinations were performed by trained physicians according to the established clinical assessment procedures. (4) Comorbidity assessment: The Charlson Comorbidity Index (CCI) was used to assess the overall burden of comorbid conditions.

### 2.3 Follow‐Up and Endpoint Definition

The date on which each patient first had both a complete respiratory muscle function assessment and a CAT score recorded was defined as the index date (baseline). From this date, all subsequent CAT scores and clinical visit records up to January 2026 were retrospectively extracted. The observation window for each patient extended to a maximum of 12 months from the index date. For patients who met the study endpoint, the event time was defined as the date of the first documented CAT score deterioration. Patients without documented deterioration were right‐censored at the date of their last valid stable‐state CAT assessment within the observation window.

The primary endpoint was defined as clinically significant CAT score deterioration, specifically the first occurrence of an increase of ≥ 2 in the stable‐state CAT score from baseline (index date) during the observation window, with all CAT scores obtained during acute exacerbation periods excluded from the analysis. This threshold corresponds to the minimal clinically important difference for the CAT. All CAT scores obtained during acute exacerbation periods were excluded from endpoint ascertainment. An acute exacerbation period was defined based on ① a documented diagnosis of acute exacerbation of COPD in the medical records or ② a record of newly initiated systemic corticosteroid and/or antibiotic therapy for acute worsening of respiratory symptoms. CAT assessments performed within 28 days of the onset of an exacerbation were considered acute‐phase assessments and were excluded from endpoint ascertainment. Patients meeting the deterioration criterion were classified as having experienced the event (event = 1), whereas those without documented deterioration were right‐censored (event = 0). Time‐to‐event was calculated from the index date to the first documented stable‐state CAT score increase of ≥ 2 points. For censored patients, follow‐up time was calculated from the index date to the last valid stable‐state CAT assessment within the observation window.

### 2.4 Data Processing

Statistical analyses were performed using R version 4.5.2, with a two‐sided *p* < 0.05 considered statistically significant. Continuous variables were compared using the independent‐samples *t*‐test, with Welch's correction when the assumption of equal variances was not met, or the Mann–Whitney *U* test, as appropriate. Candidate predictors were screened using LASSO Cox regression with 10‐fold cross‐validation, followed by multivariable Cox regression for model development. The proportional hazards assumption was assessed using Schoenfeld residuals. Internal validation was performed with 1000 bootstrap resamples, with variable selection and model fitting repeated in each resample. Model performance was evaluated using the 12‐month cumulative/dynamic time‐dependent AUC with inverse probability of censoring weighting (IPCW), calibration plots, and decision curve analysis. Incremental predictive value was assessed by comparing models with and without MIP and DTF using ΔAUC, NRI, and IDI. A sensitivity analysis was performed in patients with more regular CAT follow‐up.

## 3 Results

### 3.1 Baseline Characteristics of Stable COPD Patients

This study included 203 eligible patients with stable COPD, with a maximum follow‐up duration of 12 months. During follow‐up, 62 patients (30.5%) experienced clinically significant CAT score deterioration (deterioration group), with a median time from baseline to the first recorded deterioration of 5.50 months; the remaining 141 patients (69.5%) showed no significant deterioration (stable group). No significant between‐group differences were found in age, sex distribution, BMI, smoking status, disease duration, history of regular pulmonary rehabilitation, FEV_1_/FVC ratio, CCI, or resting DT (*p* > 0.05). Relative to the stable group, the deterioration group exhibited lower baseline FEV_1_% predicted, higher baseline CAT scores, and a higher frequency of exacerbations in the preceding year (all *p* < 0.001). Regarding respiratory muscle function indices, both MIP and DTF were markedly lower in the deterioration group versus the stable group (*p* < 0.001), and MEP was also lower in the deterioration group (*p* = 0.001; Table 1).

**TABLE 1:** Comparison of baseline clinical characteristics between the deterioration and stable groups.

Variable	Deterioration group (*N* = 62)	Stable group (*N* = 141)	*t*/*χ* ^2^/*z*	*P*
Age (years)	69.5 ± 7.9	68.0 ± 8.2	1.154	0.250
Male, *n* (%)	44 (71.0%)	109 (77.3%)	0.932	0.334
BMI (kg/m^2^)	22.3 ± 3.0	22.7 ± 3.5	−0.754	0.452
Smoking status, *n* (%)			0.342	0.843
Never smoker	15 (24.2%)	29 (20.6%)		
Former smoker	36 (58.1%)	85 (60.3%)		
Current smoker	11 (17.7%)	27 (19.1%)		
Disease duration (years)	8.1 ± 3.0	7.2 ± 3.9	1.731	0.085
FEV_1_% predicted	44.2 ± 12.9	54.3 ± 14.7	−4.690	< 0.001
FEV_1_/FVC (%)	53.0 ± 8.8	54.9 ± 8.3	−1.479	0.141
CAT score	18.9 ± 6.3	14.7 ± 4.4	4.804	< 0.001
Prior exacerbations, *n* (%)			14.742	< 0.001
0 episodes	10 (16.1%)	51 (36.2%)		
1 episode	27 (43.5%)	65 (46.1%)		
≥ 2 episodes	25 (40.3%)	25 (17.7%)		
Regular pulmonary rehabilitation history, *n* (%)	15 (24.2%)	26 (18.4%)	0.885	0.347
CCI, median (IQR)	2 (2–4)	2 (1–3)	1.290	0.197
MIP (cmH_2_O)	52.2 ± 12.7	63.5 ± 14.7	−5.270	< 0.001
MEP (cmH_2_O)	76.5 ± 16.8	85.0 ± 17.5	−3.229	0.001
DT (mm)	2.1 ± 0.5	2.1 ± 0.4	0.289	0.773
DTF (%)	23.3 ± 2.9	26.9 ± 5.4	−6.141	< 0.001

*Note:* Data are presented as mean ± standard deviation, *n* (%), or median (interquartile range, IQR).

Abbreviations: BMI, body mass index; CAT, COPD Assessment Test; CCI, Charlson Comorbidity Index; DT, diaphragm thickness; DTF, diaphragm thickening fraction; FEV_1_, forced expiratory volume in 1 s; FVC, forced vital capacity; MEP, maximal expiratory pressure; MIP, maximal inspiratory pressure.

### 3.2 Predictor Screening Using LASSO Cox Regression

Fifteen candidate predictors collected at baseline were entered into a LASSO Cox regression model for variable selection. The penalty parameter was determined by 10‐fold cross‐validation using the one‐standard‐error criterion (Figure 1A). At log(*λ*.1se) = −2.198, five variables with non‐zero coefficients were retained: FEV_1_% predicted, CAT score, prior exacerbation frequency, MIP, and DTF (Figure 1B). These variables were subsequently included in the multivariable Cox regression model.

**FIGURE 1:**
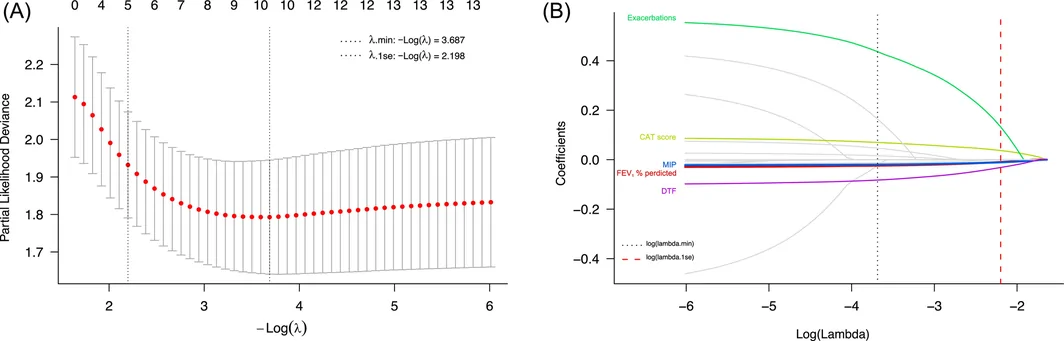
Predictor selection using LASSO Cox regression. *Note:* (A) partial likelihood deviance curve based on 10‐fold cross‐validation; (B) coefficient profiles of the 15 candidate predictors. The dashed vertical lines indicate *λ*min and *λ*.1se.

### 3.3 Multivariable Cox Regression and Nomogram Construction

Multivariable Cox regression showed that a higher frequency of prior exacerbations (HR = 1.738, 95% CI: 1.208–2.501, *p =* 0.003) and higher baseline CAT scores (HR = 1.067, 95% CI: 1.027–1.109, *p* = 0.001) were associated with a higher risk of CAT score deterioration, whereas higher FEV_1_% predicted (HR = 0.973, 95% CI: 0.956–0.990, *p* = 0.002), MIP (HR = 0.974, 95% CI: 0.957–0.992, *p* = 0.004), and DTF (HR = 0.908, 95% CI: 0.853–0.967, *p* = 0.003) were associated with a lower risk (Table 2). All variance inflation factors were < 5, indicating no substantial multicollinearity. Schoenfeld residual testing showed no evidence of violation of the proportional hazards assumption for the overall model or individual predictors. Based on the final model, a nomogram was constructed to estimate the 12‐month risk of CAT score deterioration (Figure 2).

**TABLE 2:** Multivariable Cox proportional hazards regression analysis of time to clinically significant CAT score deterioration.

Variable	*β*	SE	Wald *χ* ^2^	*P*	HR (95% CI)
FEV_1_% predicted	−0.027	0.009	9.777	0.002	0.973 (0.956–0.990)
Baseline CAT score	0.065	0.020	10.867	0.001	1.067 (1.027–1.109)
Prior exacerbations	0.553	0.186	8.860	0.003	1.738 (1.208–2.501)
MIP (cmH_2_O)	−0.026	0.009	8.094	0.004	0.974 (0.957–0.992)
DTF (%)	−0.096	0.032	8.960	0.003	0.908 (0.853–0.967)

*Note:* FEV_1_, forced expiratory volume in 1 s.

Abbreviations: *β*, regression coefficient; CAT, COPD Assessment Test; CI, confidence interval; DTF, diaphragm thickening fraction; HR, hazard ratio; MIP, maximal inspiratory pressure; SE, standard error.

**FIGURE 2:**
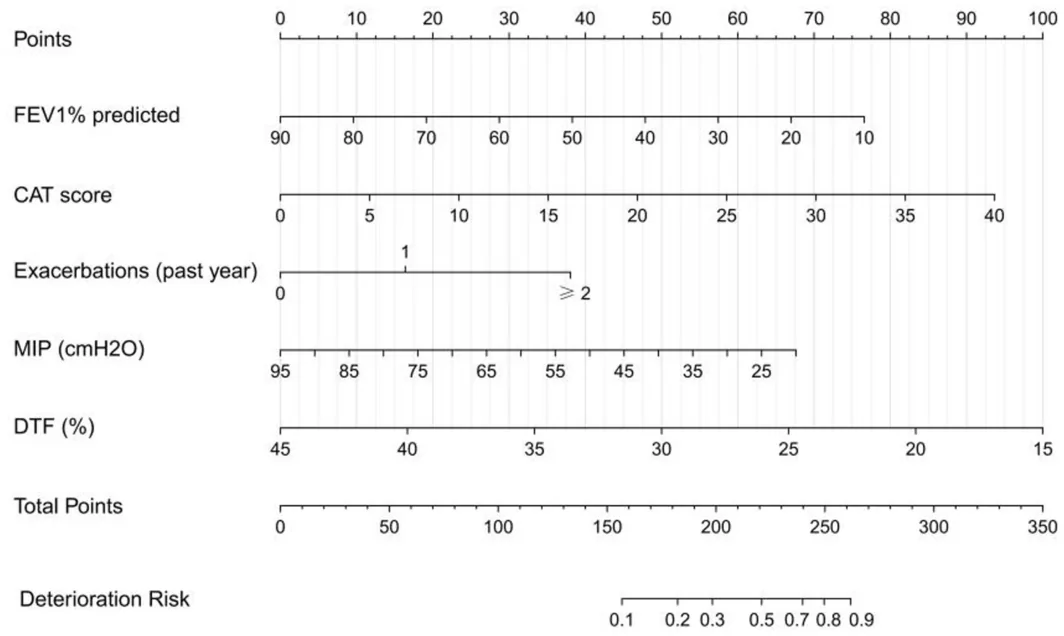
Nomogram for predicting the 12‐month risk of CAT score deterioration in patients with stable COPD. Abbreviation: COPD, chronic obstructive pulmonary disease.

### 3.4 Performance of the Prediction Model

The nomogram showed an apparent 12‐month AUC of 0.873 (95% CI: 0.822–0.924) for predicting CAT score deterioration in the development cohort (Figure 3A). After 1000 bootstrap resamples, the optimism‐corrected AUC was 0.859. The apparent calibration slope was 1.000, with a bootstrap‐corrected value of 0.922, while the corresponding calibration‐in‐the‐large values were 0.078 and 0.056, respectively. The apparent and optimism‐corrected 12‐month Brier scores were 0.131 and 0.139, respectively. The calibration curve showed good agreement between predicted and observed 12‐month deterioration probabilities, with a mean absolute error of 0.0314 (Figure 3B). The DCA indicated that the nomogram provided greater net benefit than the “treat all” and “treat none” strategies across the evaluated threshold range (Figure 3C).

**FIGURE 3:**
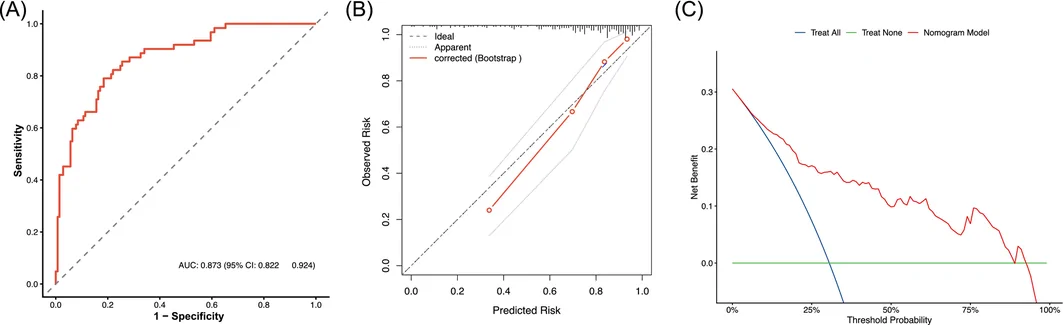
Evaluation of the prediction model. *Note:* (A) ROC curve; (B) calibration curve; (C) DCA. Abbreviations: AUC, area under the curve; CI, confidence interval; DCA, decision curve analysis; ROC, receiver operating characteristic.

### 3.5 Incremental Value of Respiratory Muscle Function

To assess the incremental predictive value of respiratory muscle function parameters, a conventional clinical model (Model 1: FEV_1_% predicted, CAT score, and prior exacerbations) was compared with an extended model additionally including MIP and DTF (Model 2). The 12‐month AUC increased from 0.801 (95% CI: 0.729–0.874) for Model 1 to 0.873 (95% CI: 0.822–0.924) for Model 2 (ΔAUC = 0.072). In addition, the inclusion of MIP and DTF yielded a continuous NRI of 0.527 (95% CI: 0.251–0.906, *p* < 0.001) and an IDI of 0.058 (95% CI: 0.019–0.136, *p* < 0.001) (Table 3). These findings suggest that MIP and DTF provided incremental predictive information beyond conventional clinical predictors in the development cohort.

**TABLE 3:** Incremental value of respiratory muscle function indices for model predictive performance.

Indices	Estimate (95% CI)	*p*
Continuous NRI (> 0)	0.527 (0.251–0.906)	< 0.001
IDI	0.058 (0.019–0.136)	< 0.001

*Note:* Model 1: FEV_1_% predicted + CAT score + prior exacerbation history; Model 2: Model 1 + MIP + DTF. Incremental values are referenced to Model 1.

Abbreviations: CAT, COPD Assessment Test; CI, confidence interval; DTF, diaphragm thickening fraction; FEV_1_, forced expiratory volume in 1 s; IDI, integrated discrimination improvement; MIP, maximal inspiratory pressure; NRI, net reclassification improvement.

### 3.6 Sensitivity Analysis

To assess the potential influence of variability in CAT assessment intervals, the five‐variable Cox model used in the primary analysis was refitted in patients with at least two valid post‐baseline stable‐state CAT assessments and a maximum interval of ≤ 6 months between consecutive assessments. This subcohort included 181 patients, of whom 55 experienced CAT score deterioration. The median number of valid follow‐up CAT assessments was 3 (IQR, 2–4), and the median interval between assessments was 4.0 months (IQR, 2.9–4.5). The estimated associations were similar in direction and magnitude to those in the primary analysis: higher FEV_1_% predicted, MIP, and DTF were associated with a lower risk of CAT score deterioration, whereas higher baseline CAT scores and more frequent prior exacerbations were associated with a higher risk (Table 4).

**TABLE 4:** Sensitivity analysis of the multivariable Cox proportional hazards model.

Variable	*β*	SE	Wald *Z*	*p*	HR (95% CI)
FEV_1_% predicted	−0.027	0.009	−3.050	0.002	0.973 (0.956–0.990)
Baseline CAT score	0.059	0.021	2.865	0.004	1.061 (1.019–1.105)
Prior exacerbations	0.499	0.192	2.605	0.009	1.647 (1.132–2.397)
MIP (cmH_2_O)	−0.026	0.010	−2.528	0.012	0.975 (0.956–0.994)
DTF (%)	−0.089	0.034	−2.639	0.008	0.915 (0.857–0.977)

Abbreviations: *β*, regression coefficient; CAT, COPD Assessment Test; CI, confidence interval; DTF, diaphragm thickening fraction; FEV_1_, forced expiratory volume in 1 s; HR, hazard ratio; MIP, maximal inspiratory pressure; SE, standard error.

In an additional sensitivity analysis excluding baseline CAT from the multivariable model, the associations of FEV_1_% predicted, prior exacerbation frequency, MIP, and DTF with CAT score deterioration remained consistent with those observed in the primary analysis. Higher FEV_1_% predicted (HR = 0.970, 95% CI: 0.954–0.987), MIP (HR = 0.969, 95% CI: 0.952–0.985), and DTF (HR = 0.890, 95% CI: 0.836–0.947) remained associated with a lower risk of CAT score deterioration, whereas more frequent prior exacerbations remained associated with a higher risk (HR = 1.684, 95% CI: 1.181–2.403; all *p* ≤ 0.004).

## 4 Discussion

In this retrospective cohort of stable COPD patients, a nomogram including respiratory muscle function parameters was constructed and internally validated. The results demonstrated that higher FEV_1_% predicted, MIP, and DTF were independently associated with a lower risk of CAT score deterioration, whereas higher baseline CAT scores and prior exacerbation frequency were associated with a higher risk. Adding MIP and DTF to conventional clinical predictors increased model discrimination and reclassification metrics in the development cohort, suggesting that respiratory muscle function may provide supplementary predictive information beyond traditional clinical assessment.

Traditionally, risk evaluation and prognostication in patients with stable COPD have relied heavily on the degree of airflow limitation. However, in clinical practice, patients with comparable pulmonary function impairment often show notable heterogeneity in their symptom and health‐status trajectories [7, 12]. The findings of this study suggest that respiratory muscle function indices may provide supplementary predictive information beyond conventional airflow limitation measures. In patients with COPD, chronic increases in airway resistance, pulmonary hyperinflation, and systemic inflammation place the respiratory muscles, particularly the diaphragm, in a state of sustained mechanical disadvantage with high load and reduced efficiency [13]. DTF measured by bedside ultrasound is a non‐invasive ultrasound‐derived measure that may reflect aspects of diaphragmatic contractile activity [14], whereas MIP is an effort‐dependent measure commonly used to assess global inspiratory muscle strength [15]. Lower MIP and DTF may therefore be consistent with reduced inspiratory muscle performance and diaphragmatic functional reserve, although these measures do not directly quantify these physiological properties in isolation. Such functional impairment, if present, could contribute to reduced capacity to meet increased ventilatory demands during daily activity or physiological stress and thereby be associated with greater dyspnea or exercise limitation. Previous studies have reported associations of respiratory muscle dysfunction with exercise limitation and dyspnea in stable COPD [16]. In the present cohort, lower MIP and DTF were associated with subsequent CAT score deterioration, supporting their potential utility as complementary risk markers rather than establishing a direct mechanistic relationship.

It is noteworthy that although MEP differed between the deterioration and stable groups, it was not retained by LASSO Cox regression. This finding may reflect the limited additional predictive information provided by MEP after accounting for the other selected variables. Furthermore, from a clinical pathophysiological standpoint, pulmonary hyperinflation and increased ventilatory loading in COPD predominantly affect the mechanical efficiency of the inspiratory muscles [17], whereas expiratory muscles may be less affected during quiet breathing or low‐intensity activity in the stable state. Inspiratory muscle measures may therefore have a stronger association with subsequent CAT score changes than expiratory muscle measures [18]. MIP provides an effort‐dependent estimate of global inspiratory muscle strength and may also be influenced by patient cooperation and systemic muscle condition, whereas DTF provides ultrasound‐derived information on diaphragmatic thickening during inspiration but may be influenced by loading conditions and measurement technique [19]. The combined use of these two measures may provide complementary perspectives on inspiratory muscle function status.

The nomogram developed in this study may provide additional information for risk assessment by integrating conventional clinical indicators with respiratory muscle function. Previous evidence suggests that breathing exercises can improve respiratory symptoms and functional outcomes in patients with COPD [20]. Specific inspiratory muscle training has also been shown to improve inspiratory muscle performance when incorporated into rehabilitation programs [21]. These findings support further investigation of respiratory muscle function as a potential marker for identifying patients who may benefit from closer assessment or targeted rehabilitation. However, the present model requires external validation before clinical application.

Several limitations should be considered when interpreting the present findings. First, the single‐center retrospective design and limited sample size may restrict the generalizability of the model, and external validation in independent populations is still required. Detailed cumulative smoking exposure (pack‐years) was not consistently documented in the retrospective medical records and therefore could not be evaluated. In addition, predictor selection based on LASSO regression may be influenced by the characteristics of the development sample, although bootstrap resampling was used for internal validation. Second, MIP is effort dependent, and DTF measurements may be affected by operator experience and image acquisition; despite standardized procedures and repeated measurements, residual measurement variability cannot be excluded. Third, only baseline respiratory muscle parameters were available, and longitudinal changes in respiratory muscle function, dynamic hyperinflation, absolute lung volumes, and exercise‐related physiological measures were not assessed. In addition, longitudinal information on changes in maintenance pharmacotherapy and participation in pulmonary rehabilitation or inspiratory muscle training during follow‐up was not consistently available in the retrospective records and therefore could not be incorporated into the analysis. These post‐baseline interventions may have influenced subsequent CAT trajectories and represent a potential source of residual treatment‐related confounding. Baseline CAT score was also included as a predictor while serving as the reference value for defining subsequent CAT score deterioration, which should be considered when interpreting its association with the outcome. Finally, CAT assessments were obtained during routine clinical visits rather than at fixed intervals, resulting in heterogeneous assessment schedules and potential interval‐censoring or informative follow‐up bias. Although sensitivity analysis in patients with more regular CAT follow‐up yielded similar estimates, residual bias may remain.

## 5 Conclusions

In this retrospective cohort of patients with stable COPD, lower MIP and DTF were independently associated with a higher risk of CAT score deterioration. Incorporating these parameters into a model with conventional clinical predictors provided additional predictive information in the development cohort. These findings support further evaluation of respiratory muscle function for risk assessment in stable COPD, although external validation is required before clinical application.

## Author Contributions

**Muye Niu:** conceptualization, methodology, data curation, investigation, writing – original draft. **Changye Wu:** investigation, validation, writing – review and editing. **Shaoting Geng:** formal analysis, software, writing – review and editing. **Mian Song:** investigation, resources, writing – review and editing. **Mingchao Cao:** supervision, project administration, funding acquisition, writing – review and editing. All authors have read and approved the final version of the manuscript.

## Funding

This study was supported by the Hebei Provincial Health Commission Research Project (No. 20260316).

## Ethics Statement

The study was approved by the Ethics Committee of the Second Hospital of Hebei Medical University (Approval No.: 2025‐R391‐N1) and was conducted in accordance with the Declaration of Helsinki. Given the retrospective nature of the study and the use of anonymized clinical data, the requirement for informed consent was waived by the Ethics Committee.

## Conflicts of Interest

The authors declare no conflicts of interest.

## Data Availability

The data used and/or analyzed during the current study are available from the corresponding author.
